# Supplementary material for: Small nucleoli are a cellular hallmark of longevity
Source: Nat Commun. 2017 Aug 30;8:16083. doi: 10.1038/ncomms16083 (PMC5582349; doi:10.1038/ncomms16083)
Supplement: Supplementary Information [file ncomms16083-s1.pdf]

Title of file for HTML: Peer Review File
Description:

Title of file for HTML: Supplementary Information
Description: Supplementary Figures, Supplementary Tables.

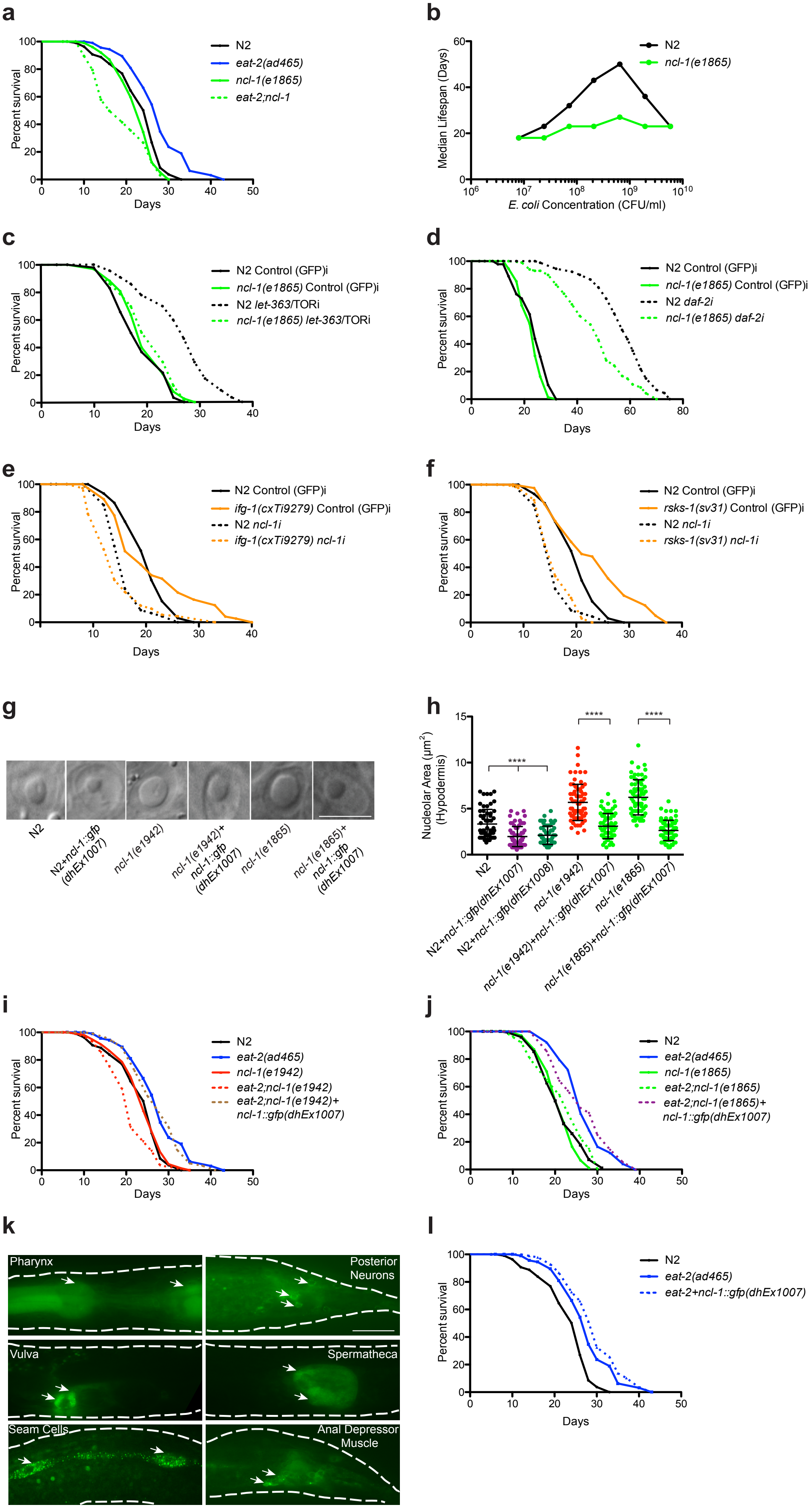

a
Percent survival
Days
N2
eat-2(ad465)
ncl-1(e1865)
eat-2;ncl-1
b
Median Lifespan (Days)
E. coli Concentration (CFU/ml)
N2
ncl-1(e1865)
c
Percent survival
Days
N2 Control (GFP)i
ncl-1(e1865) Control (GFP)i
N2 let-363/TORi
ncl-1(e1865) let-363/TORi
d
Percent survival
Days
N2 Control (GFP)i
ncl-1(e1865) Control (GFP)i
N2 daf-2i
ncl-1(e1865) daf-2i
e
Percent survival
Days
N2 Control (GFP)i
ifg-1(cxTi9279) Control (GFP)i
N2 ncl-1i
ifg-1(cxTi9279) ncl-1i
f
Percent survival
Days
N2 Control (GFP)i
rsks-1(sv31) Control (GFP)i
N2 ncl-1i
rsks-1(sv31) ncl-1i
g
N2
N2+ncl-1::gfp (dhEx1007)
ncl-1(e1942)
ncl-1(e1942)+ ncl-1::gfp (dhEx1007)
ncl-1(e1865)
ncl-1(e1865)+ ncl-1::gfp (dhEx1007)
h
Nucleolar Area (μm²) (Hypodermis)
****
N2
N2+ncl-1::gfp(dhEx1007)
N2+ncl-1::gfp(dhEx1008)
ncl-1(e1942)
ncl-1(e1942)+ncl-1::gfp(dhEx1007)
ncl-1(e1865)
ncl-1(e1865)+ncl-1::gfp(dhEx1007)
i
Percent survival
Days
N2
eat-2(ad465)
ncl-1(e1942)
eat-2;ncl-1(e1942)
eat-2;ncl-1(e1942)+ ncl-1::gfp(dhEx1007)
j
Percent survival
Days
N2
eat-2(ad465)
ncl-1(e1865)
eat-2;ncl-1(e1865)
eat-2;ncl-1(e1865)+ ncl-1::gfp(dhEx1007)
k
Pharynx
Posterior Neurons
Vulva
Spermatheca
Seam Cells
Anal Depressor Muscle
l
Percent survival
Days
N2
eat-2(ad465)
eat-2+ncl-1::gfp(dhEx1007)

Supplementary Fig. 1. (a) Longevity of *eat-2(ad465)* is abolished with the loss of *ncl-1(e1865)* (P<0.0001). (b) *ncl-1(e1865)* is significantly shorter lived than N2 upon bacterial dilution across 7 different concentrations (P<0.0001). (c,d) *ncl-1(e1865)* is shorter lived than N2 upon *let-363/*TOR and *daf-2* RNAi (P<0.0001) (e,f) *ncl-1* RNAi abrogates the longevity of *ifg-1(cxTi9279)* (P<0.0001) and *rsks-1(sv31)* (P<0.0001). (g,h,i,j) Over-expression of *ncl-1 (+)* decreases nucleolar size in N2. Restoration of *ncl-1* transgenically rescues nucleolar size and longevity in *ncl-1* and *eat-2;ncl-1* animals respectively (****P<0.0001, unpaired t-test and lifespan analysis P<0.0001, log-rank test). Scale bar represents 5 µm. (k) *ncl-1* is expressed in multiple tissues. Scale bar represents 20 µm. (l) Over-expression of *ncl-1 (+)* does not extend longevity of *eat-2(ad465)* further (*eat-2* vs *eat-2+ncl-1::gfp(dhEx1007)* P=0.135, log-rank test).

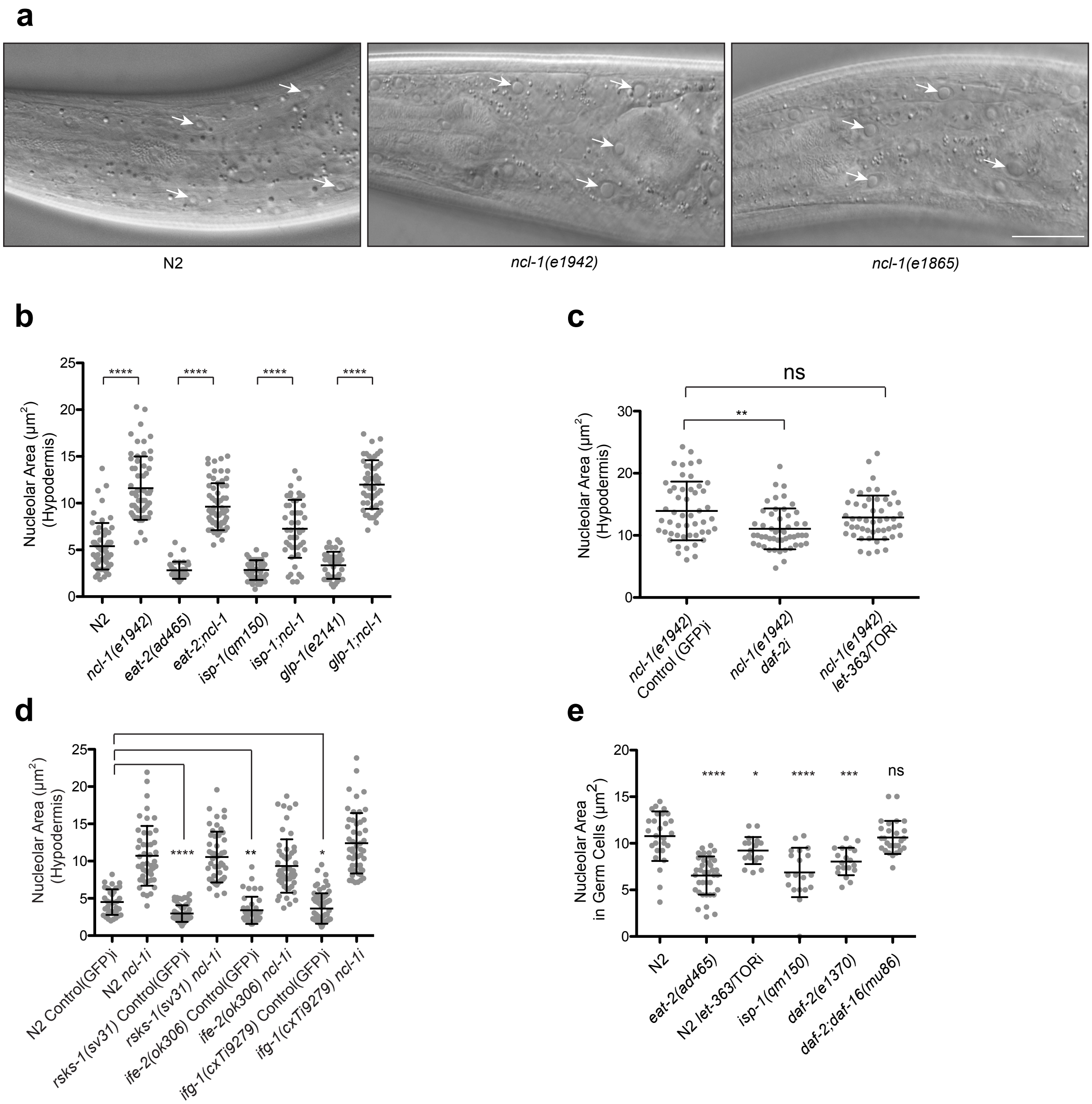

Supplementary Fig. 2. (a) *ncl-1* mutants possess larger nucleoli compared to N2. Hypodermal nucleoli are depicted here (b,c) Small nucleoli of *eat-2(ad465)*, *isp-1(qm150)*, *glp-1(e2141)* and *daf-2i* become enlarged with the loss of *ncl-1* but are still significantly smaller than *ncl-1* single mutants with the exception of *let-363/*TOR knockdown. (d) Long-lived *rsks-1(sv31)* (P<0.0001)*, ife-2(ok306)* (P=0.008) and *ifg-1(cxTi9279)* (P=0.030) on control RNAi have smaller nucleoli compared to N2 on control RNAi. This effect is reversed by the loss of *ncl-1,* which invariably enlarges the nucleolar size of all the strains. (e) Long-lived *eat-2(ad465)*, *let-363/*TOR RNAi, *isp-1(qm150)* and *daf-2(e1370)* animals possess small nucleoli in germ cells compared to N2 while short-lived *daf-2;daf-16* animals possess nucleoli similar to N2. Error bars represent mean ± SD * P<0.05, ** P<0.01, *** P<0.001, **** P<0.0001, ns: non-significant, unpaired t-test. Scale bar represents 20 μm.

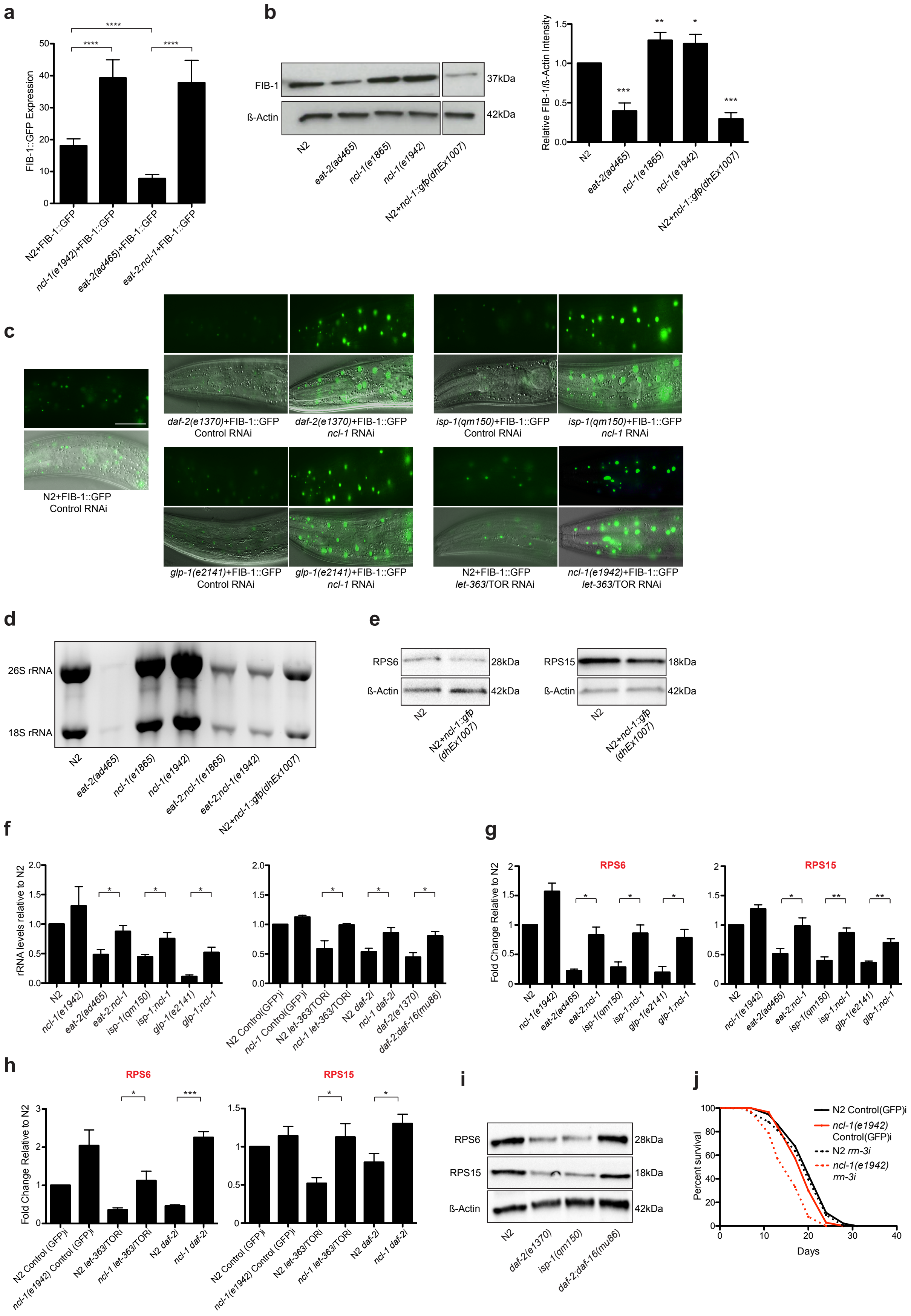
a
FIB-1::GFP Expression
N2+FIB-1::GFP
ncl-1(e1942)+FIB-1::GFP
eat-2(ad465)+FIB-1::GFP
eat-2;ncl-1+FIB-1::GFP
****
b
FIB-1
ß-Actin
37kDa
42kDa
N2
eat-2(ad465)
ncl-1(e1865)
ncl-1(e1942)
N2+ncl-1::gfp(dhEx1007)
Relative FIB-1/ß-Actin Intensity
c
N2+FIB-1::GFP
Control RNAi
daf-2(e1370)+FIB-1::GFP
Control RNAi
daf-2(e1370)+FIB-1::GFP
ncl-1 RNAi
isp-1(qm150)+FIB-1::GFP
Control RNAi
isp-1(qm150)+FIB-1::GFP
ncl-1 RNAi
glp-1(e2141)+FIB-1::GFP
Control RNAi
glp-1(e2141)+FIB-1::GFP
ncl-1 RNAi
N2+FIB-1::GFP
let-363/TOR RNAi
ncl-1(e1942)+FIB-1::GFP
let-363/TOR RNAi
d
26S rRNA
18S rRNA
N2
eat-2(ad465)
ncl-1(e1865)
ncl-1(e1942)
eat-2;ncl-1(e1865)
eat-2;ncl-1(e1942)
N2+ncl-1::gfp(dhEx1007)
e
RPS6
ß-Actin
28kDa
42kDa
RPS15
18kDa
N2
N2+ncl-1::gfp
(dhEx1007)
f
rRNA levels relative to N2
N2
ncl-1(e1942)
eat-2(ad465)
eat-2;ncl-1
isp-1(qm150)
isp-1;ncl-1
glp-1(e2141)
glp-1;ncl-1
N2 Control(GFP)i
ncl-1 Control(GFP)i
N2 let-363/TORi
ncl-1 let-363/TORi
N2 daf-2i
ncl-1 daf-2i
daf-2(e1370)
daf-2;daf-16(mu86)
g
RPS6
RPS15
Fold Change Relative to N2
h
RPS6
RPS15
Fold Change Relative to N2
N2 Control (GFP)i
ncl-1(e1942) Control (GFP)i
N2 let-363/TORi
ncl-1 let-363/TORi
N2 daf-2i
ncl-1 daf-2i
i
RPS6
RPS15
ß-Actin
28kDa
18kDa
42kDa
N2
daf-2(e1370)
isp-1(qm150)
daf-2;daf-16(mu86)
j
Percent survival
Days
N2 Control(GFP)i
ncl-1(e1942)
Control(GFP)i
N2 rrn-3i
ncl-1(e1942)
rrn-3i

Supplementary Fig. 3. (a,b) FIB-1 is strongly up-regulated in *ncl-1* and *eat-2;ncl-1* and down-regulated in *eat-2(ad465)* and in worms with *ncl-1 (+)* over-expression. (c) FIB-1 levels are reduced in long-lived *daf-2(e1370)*, *isp-1(qm150)*, *glp-1(e2141)* and *let-363/*TOR RNAi animals and this effect is lost with the loss of *ncl-1.* Scale bar represents 20 μm. (d) RNA gel showing higher levels of rRNA in *ncl-1* mutants and reduced levels in *eat-2(ad465)* and in *ncl-1 (+)* over-expressing worms (e) *ncl-1 (+)* over-expression decreases RPS6 and RPS15 levels. (f,g,h) Quantification of rRNA, RPS6 and RPS15 levels relative to N2 (i) RPS6 and RPS15 levels are reduced in long-lived *isp-1(qm150)* and *daf-2(e1370)* and increased in *daf-2;daf-16(mu86).* (j) Egg-on *rrn-3i* does not affect the lifespan of N2 whereas it induces a modest shortening of lifespan in *ncl-1(e1942).* Error bars represent mean ± SEM * $P<0.05$, ** $P<0.01$, *** $P<0.001$, **** $P<0.0001$, unpaired t-test and one-way anova

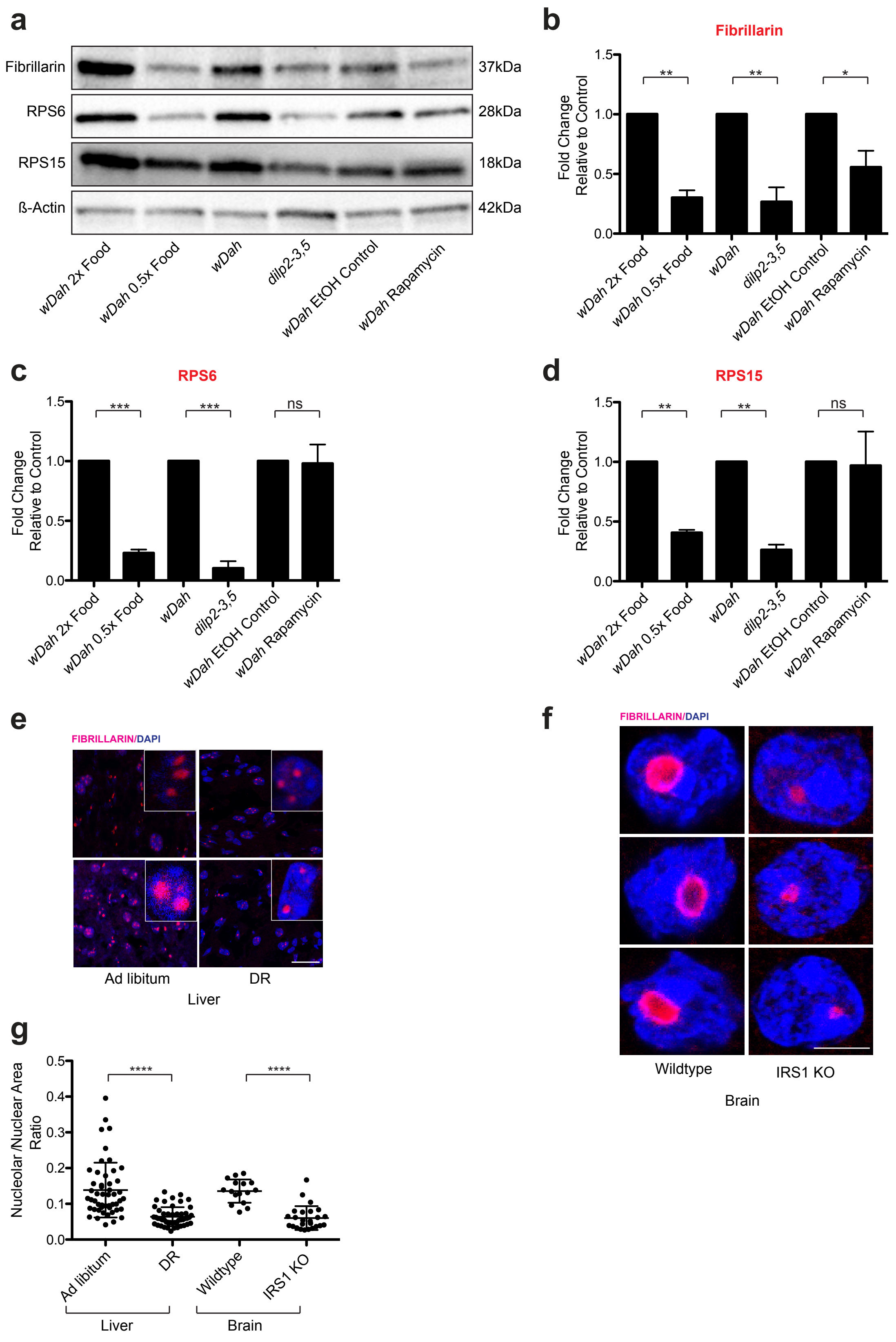

a
Fibrillarin
RPS6
RPS15
ß-Actin
37kDa
28kDa
18kDa
42kDa
wDah 2x Food
wDah 0.5x Food
wDah
dilp2-3,5
wDah EtOH Control
wDah Rapamycin
b
Fibrillarin
Fold Change Relative to Control
**
**
*
c
RPS6
***
***
ns
d
RPS15
**
**
ns
e
FIBRILLARIN/DAPI
Ad libitum
DR
Liver
f
FIBRILLARIN/DAPI
Wildtype
IRS1 KO
Brain
g
Nucleolar/Nuclear Area Ratio
****
****
Ad libitum
DR
Wildtype
IRS1 KO
Liver
Brain

Supplementary Fig. 4. (a,b,c,d) *Drosophila* subjected to DR and long-lived *dilp2-3,5* mutants exhibit reduced FIB-1, RPS6 and RPS15 levels. Rapamycin treatment reduces FIB-1 levels without affecting RPS6 and RPS15. (e,f,g) Mice subjected to DR and long-lived IRS1 knockout mice exhibit smaller nucleoli compared to ad libitum fed mice and wildtype controls in liver and brain. Error bars represent mean ± SEM (b,c,d) and mean ± SD (g). Scale bars represent 10 μm (e) and 5 μm (f). * $P<0.05$, ** $P<0.01$, *** $P<0.001$, **** $P<0.0001$, unpaired t-test.

Fig. 3c

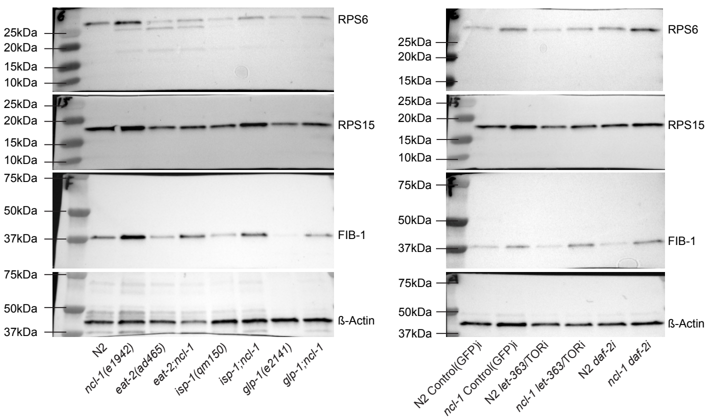

Supplementary Fig. 3b

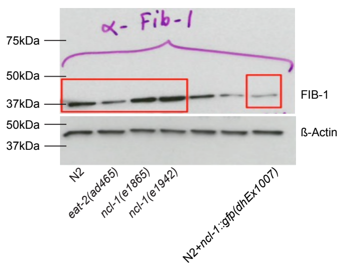

Supplementary Fig. 3e

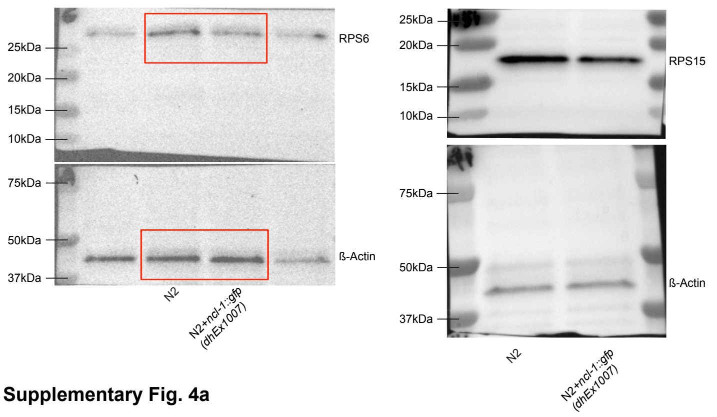

Supplementary Fig. 3i

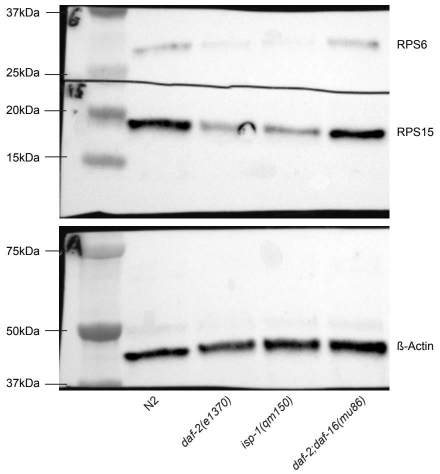

Supplementary Fig. 4a

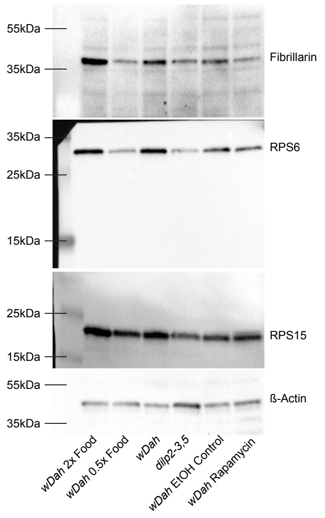

Supplementary Fig. 5. Uncropped western blot images

Supplementary Table 1: Lifespan Analyses

Strain/Treatment	Median Lifespan	Median Difference from Control (%)	Worms*	P value	Reference Control.
N2	26		91/120		
eat-2(ad465)	28	+7.69	68/120	<0.0001	vs N2
ncl-1(e1942)	24	-7.69	75/120	ns	vs N2
ncl-1(e1865)	24	-7.69	98/120	0.03	vs N2
eat-2;ncl-1(e1942)	21	-25	40/120	<0.0001	vs *eat-2*
eat-2;ncl-1(e1865)	16	-42.85	72/120	<0.0001	vs *eat-2*
N2	25		100/120		
eat-2(ad465)	30	+20	48/120	0.002	vs N2
ncl-1(e1942)	25	0	83/120	ns	vs N2
ncl-1(e1865)	25	0	91/120	ns	vs N2
eat-2;ncl-1(e1942)	25	-16.66	43/120	<0.0001	vs *eat-2*
eat-2;ncl-1(e1865)	17	-43.33	79/120	<0.0001	vs *eat-2*
N2	24		89/120		
eat-2(ad465)	27	+12.5	64/120	0.002	vs N2
ncl-1(e1942)	24	0	82/120	ns	vs N2
ncl-1(e1865)	24	0	79/120	ns	vs N2
eat-2;ncl-1(e1942)	24	-12.5	58/120	<0.0001	vs *eat-2*
eat-2;ncl-1(e1865)	17	-37.07	94/120	<0.0001	vs *eat-2*
BDR					
N2 (5.85×10^9CFU/ml)	23		76/90		
N2 (1.95×10^9CFU/ml)	36	+56.52	83/90	<0.0001	vs 5.85×10^9
N2 (6.5×10^8CFU/ml)	50	+38.88	82/90	<0.0001	vs 1.95×10^9
N2 (2.1×10^8CFU/ml)	43	-14	89/90	<0.0001	vs 6.5×10^8
N2 (7.2×10^7CFU/ml)	32	-25.50	86/90	<0.0001	vs 2.1×10^8
N2 (2.4×10^7CFU/ml)	23	-28.12	84/90	<0.0001	vs 7.2×10^7
N2 (8×10^6CFU/ml)	18	-21.73	81/90	0.0003	vs 2.4×10^7

ncl-1(e1942) (5.85×10^9CFU/ml)	23	0	58/90	ns	vs N2 5.85×10^9
ncl-1(e1942) (1.95×10^9CFU/ml)	23	-36.11	76/90	<0.0001	vs N2 1.95×10^9
ncl-1(e1942) (6.5×10^8CFU/ml)	32	-36	85/90	<0.0001	vs N2 6.5×10^8
ncl-1(e1942) (2.1×10^8CFU/ml)	27	-37.20	88/90	<0.0001	vs N2 2.1×10^8
ncl-1(e1942) (7.2×10^7CFU/ml)	23	-28.12	83/90	<0.0001	vs N2 7.2×10^7
ncl-1(e1942) (2.4×10^7CFU/ml)	18	-21.73	84/90	0.0092	vs N2 2.4×10^7
ncl-1(e1942) (8×10^6CFU/ml)	13	-27.77	86/90	ns	vs N2 8×10^6
ncl-1(e1865) (5.85×10^9CFU/ml)	23	0	77/90	ns	vs N2 5.85×10^9
ncl-1(e1865) (1.95×10^9CFU/ml)	23	-36.11	85/90	<0.0001	vs N2 1.95×10^9
ncl-1(e1865) (6.5×10^8CFU/ml)	27	-46	78/90	<0.0001	vs N2 6.5×10^8
ncl-1(e1865) (2.1×10^8CFU/ml)	23	-46.51	79/90	<0.0001	vs N2 2.1×10^8
ncl-1(e1865) (7.2×10^7CFU/ml)	23	-28.12	86/90	<0.0001	vs N2 7.2×10^7
ncl-1(e1865) (2.4×10^7CFU/ml)	18	-21.73	75/90	0.0253	vs N2 2.4×10^7
ncl-1(e1865) (8×10^6CFU/ml)	18	0	87/90	ns	vs N2 8×10^6
N2 GFPi	21		94/120		
N2 *let-363/*TORi	28	+33.33	82/120	<0.0001	vs N2 GFPi
ncl-1(e1942) GFPi	26		79/120		
*ncl-1(e1942) let-363/*TORi	23	-17.85	82/120	0.0046	vs N2 *let-363/*TORi
ncl-1(e1865) GFPi	23		92/120		
*ncl-1(e1865) let-363/*TORi	26	-7.14	83/120	<0.0001	vs N2 *let-363/*TORi
N2 GFPi	24		90/120		
N2 *let-363/*TORi	29	+20.83	65/120	<0.0001	vs N2 GFPi
ncl-1(e1942) GFPi	22		45/120		
*ncl-1(e1942) let-363/*TORi	27	-6.89	79/120	<0.0001	vs N2 *let-363/*TORi
ncl-1(e1865) GFPi	22		65/120		
*ncl-1(e1865) let-363/*TORi	24	-17.24	92/120	<0.0001	vs N2 *let-363/*TORi

N2 GFPi	19		81/120		
N2 *let-363/*TORi	27	+42.10	60/120	<0.0001	vs N2 GFPi
ncl-1(e1942) GFPi	17		54/120		
*ncl-1(e1942) let-363/*TORi	15	-44.44	49/120	<0.0001	vs N2 *let-363/*TORi
ncl-1(e1865) GFPi	19		90/120		
*ncl-1(e1865) let-363/*TORi	23	-14.81	78/120	<0.0001	vs N2 *let-363/*TORi
N2 GFPi	25		99/120		
N2 *daf-2i*	55	+120	83/120	<0.0001	vs N2 GFPi
ncl-1(e1942) GFPi	21		84/120		
ncl-1(e1942) daf-2i	34	-38.18	77/120	<0.0001	vs N2 *daf-2i*
ncl-1(e1865) GFPi	21		104/120		
ncl-1(e1865) daf-2i	45	-18.18	42/120	<0.0001	vs N2 *daf-2i*
N2 GFPi	20		87/120		
N2 *daf-2i*	58	+190	86/120	<0.0001	vs N2 GFPi
ncl-1(e1942) GFPi	20		64/120		
ncl-1(e1942) daf-2i	51	-12.06	58/120	<0.0001	vs N2 *daf-2i*
ncl-1(e1865) GFPi	20		94/120		
ncl-1(e1865) daf-2i	47	-18.96	79/120	<0.0001	vs N2 *daf-2i*
N2 GFPi	24		62/120		
N2 *daf-2i*	59	+145	69/120	<0.0001	vs N2 GFPi
ncl-1(e1942) GFPi	24		31/120		
ncl-1(e1942) daf-2i	48	-18.64	47/120	<0.0001	vs N2 *daf-2i*
ncl-1(e1865) GFPi	24		74/120		
ncl-1(e1865) daf-2i	48	-18.64	73/120	<0.0001	vs N2 *daf-2i*
N2	22		66/120		
ncl-1(e1942)	20		64/120		
glp-1(e2141)	33	+50	102/120	<0.0001	vs N2
glp-1;ncl-1	22	-33.33	92/120	<0.0001	vs *glp-1*
N2	24		83/120		
ncl-1(e1942)	24		57/120		
glp-1(e2141)	40	+66.66	102/120	<0.0001	vs N2

glp-1;ncl-1	24	-40	92/120	<0.0001	vs *glp-1*
glp-1(e2141)	37		76/120	<0.0001	
glp-1;ncl-1	25	-32.43	103/120	<0.0001	vs *glp-1*
N2	23		93/120		
ncl-1(e1942)	23		89/120		
isp-1(qm150)	29	+26.08	39/120	<0.0001	vs N2
isp-1;ncl-1	25	-13.79	103/120	0.002	vs *isp-1*
N2	23		93/120		
ncl-1(e1942)	23		89/120		
isp-1(qm150)	34	+47.82	38/120	<0.0001	vs N2
isp-1;ncl-1	26	-23.52	69/120	0.0016	vs *isp-1*
isp-1(qm150)	34		29/120	<0.0001	
isp-1;ncl-1	22	-35.29	70/120	<0.0001	vs *isp-1*
N2	24		86/120		
N2+*ncl-1::gfp (dhEx1007)*	29	+20.83	78/120	<0.0001	vs N2
N2+*ncl-1::gfp (dhEx1008)*	27	+12.50	63/120	<0.0001	vs N2
N2	16		78/120		
N2+*ncl-1::gfp (dhEx1007)*	22	+37.50	57/120	<0.0001	vs N2
N2	17		63/120		
N2+*ncl-1::gfp (dhEx1007)*	23	+35.29	48/120	<0.0001	vs N2
N2	26		91/120		
eat-2(ad465)	28	+7.69	68/120	<0.0001	vs N2
ncl-1(e1942)	24		75/120		
eat-2;ncl-1	21	-25	40/120	<0.0001	vs *eat-2*
eat-2;ncl-1+ ncl-1::gfp(dhEx1007)	27	+3.57	78/120	ns	vs *eat-2*

eat-2+ncl-1::gfp (dhEx1007)	30	+7.14	62/120	ns	vs *eat-2*
N2	20		87/120		
eat-2(ad465)	26	+30	66/120	<0.0001	vs N2
ncl-1(e1865)	20		94/120		
eat-2;ncl-1	23	-11.53	77/120	<0.0001	vs *eat-2*
eat-2;ncl-1+ ncl-1::gfp(dhEx1007)	26	0	56/120	ns	vs *eat-2*
N2 GFPi	24		90/120		
N2 *fib-1i*	27	+12.50	56/120	<0.0001	vs N2 GFPi
N2 GFPi	19		81/120		
N2 *fib-1i*	23	+21.10	47/120	0.0004	vs N2 GFPi
N2 GFPi	19		81/120		
N2 *fib-1i*	29	+52.63	87/120	<0.0001	vs N2 GFPi
N2 GFPi	24		89/120		
N2 *fib-1i*	29	+20.83	87/120	<0.0001	vs N2 GFPi
N2 GFPi	24		90/120		
N2 *fib-1i*	26	+8.33	75/120	0.0004	vs N2 GFPi
N2 GFPi	18		95/120		
N2 *ncl-1i*	16	-11.11	68/120	0.0167	vs N2 GFPi
ife-2(ok306) GFPi	22	+22.22	86/120	<0.0001	vs N2 GFPi
ife-2(ok306) ncl-1i	18	-22.22	68/120	<0.0001	vs *ife-2* GFPi
N2 GFPi	19		105/120		
N2 *ncl-1i*	17	-10.52	51/120	<0.0001	vs N2 GFPi
ife-2(ok306) GFPi	23	+21.05	94/120	<0.0001	vs N2 GFPi
ife-2(ok306) ncl-1i	15	-34.78	73/120	<0.0001	vs *ife-2* GFPi
N2 GFPi	18		93/120		
N2 *ncl-1i*	15	-16.66	64/120	0.0180	vs N2 GFPi

rsks-1(sv31) GFPi	22	+22.22	68/120	0.0053	vs N2 GFPi
rsks-1(sv31) ncl-1i	18	-22.22	72/120	0.0077	vs *rsks-1* GFPi
N2 GFPi	21		101/120		
N2 *ncl-1i*	16	-23.80	47/120	<0.0001	vs N2 GFPi
rsks-1(sv31) GFPi	21		61/120	<0.0001	vs N2 GFPi
rsks-1(sv31) ncl-1i	16	-23.80	56/120	<0.0001	vs *rsks-1* GFPi
N2 GFPi	21		101/120		
N2 *ncl-1i*	16	-23.80	47/120	<0.0001	vs N2 GFPi
ifg-1(cxTi9279) GFPi	19		84/120	<0.0001	vs N2 GFPi
ifg-1(cxTi9279) ncl-1i	14	-26.31	94/120	<0.0001	vs *ifg-1* GFPi
N2 GFPi	20		100/120		
ncl-1i(e1942) GFPi	20		76/120		vs N2 GFPi
N2 *rrn-3i*	20	0	59/120	0.3825	vs N2 GFPi
ncl-1i(e1942) rrn-3i	17	-15	78/120	<0.0001	vs *ncl-1* GFPi

* no. of dead worms/total no. of worms analyzed
p-values for statistical analyses were calculated using Mantel-Cox Log Rank test.
Worms that escaped the dishes, had internal hatching or had bursting of vulva were censored from the experiment.

Supplementary Table 2: Nucleolar Size Analyses

Strain/Condition	Average nucleolar area (µm^2)	Standard Deviation	% Change relative to N2 or N2 Control (GFP) RNAi
**Nucleoli in hypodermis**			
N2	5.390	± 2.480	
eat-2(ad465)	2.821	± 0.919	-47.65
N2 *let-363*/TORi	2.835	± 1.141	-47.40
isp-1(qm150)	2.845	± 1.053	-47.22
glp-1(e2141)	3.354	± 1.447	-37.76
daf-2(e1370)	2.321	± 1.032	-56.92
daf-2;daf-16(mu86)	4.583	± 1.965	-14.98
N2 GFPi	4.492	± 1.150	
N2 *fib-1i*	2.131	± 0.779	-52.56
ifg-1(cxTi9279) GFPi	3.640	± 2.03	-18.96
ife-2(ok306) GFPi	3.400	± 1.823	-24.30
rsks-1(sv31) GFPi	2.959	± 1.118	-34.12
**Nucleoli in pharyngeal muscle**			
N2	2.017	± 0.562	
eat-2(ad465)	1.581	± 0.481	-21.60
N2 *let-363*/TORi	1.719	± 0.455	-14.79
isp-1(qm150)	1.222	± 0.350	-39.42
glp-1(e2141)	1.669	± 0.466	-17.25
daf-2(e1370)	1.555	± 0.426	-22.91
daf-2;daf-16(mu86)	2.240	± 0.668	+11.05

Nucleoli in germ cells

N2	10.769	± 2.657	
eat-2(ad465)	6.549	± 2.050	-39.18
N2 *let-363*/TORi	9.227	± 1.451	-14.32
isp-1(qm150)	7.264	± 2.119	-32.54
daf-2(e1370)	8.047	± 1.471	-25.27
daf-2;daf-16(mu86)	10.633	± 1.779	-1.269

BDR

Strain and Condition	Average nucleolar area (μm^2)	Standard Deviation	% Change relative to N2 ($5.85x10^9$)
N2 ($5.85x10^9$)	4.218	± 1.725	
N2 ($1.96\ x10^9$)	2.757	± 0.988	- 34.61
N2 ($6.50\ x10^8$)	1.531	± 0.365	- 63.70

Supplementary Table 3: Characteristics of human volunteers following lifestyle intervention

	**Growing Old Together participants (N=10)**
Age, years	62.4 (4.1)
Females, N (%)	5 (50)
Lipid lowering medication, N (%)	3 (30)
Antihypertensive medication, N (%)	3 (30)
Intervention parameters	
Baseline weight, kg	81.0 (12.8)
After intervention weight, kg	74.2 (12.1)
Baseline glucose, mmol/L	5.1 (0.4)
After intervention glucose, mmol/L	4.9 (0.5)
Baseline SBP, mmHg	146.5 (14.9)
After intervention SBP, mmHg	136.4 (9.4)
Baseline BMI, kg/m^2	28.4 (2.7)
After intervention BMI, kg/m^2	26.0 (2.4)

Means (standard deviations) are provided unless otherwise stated.
